# Supplementary material for: StrainFacts accurately quantifies both endogenous and live biotherapeutic product strain abundances in simulated and clinical vaginal microbiota samples
Source: bioRxiv. 2025 Aug 21:2025.08.15.670563. Preprint. [Version 1] doi: 10.1101/2025.08.15.670563 (PMC12393323; doi:10.1101/2025.08.15.670563)
Supplement: Supplement 1 [file NIHPP2025.08.15.670563v1-supplement-1.pdf]

bioRxiv preprint doi: https://doi.org/10.1101/2025.08.15.670563; this version posted August 21, 2025. The copyright holder for this preprint (which was not certified by peer review) is the author/funder, who has granted bioRxiv a license to display the preprint in perpetuity. It is made available under aCC-BY 4.0 International license.

Supplemental Figures

Figure S1. *Lactobacillus crispatus* LBP strain quantification using a StrainFacts workflow

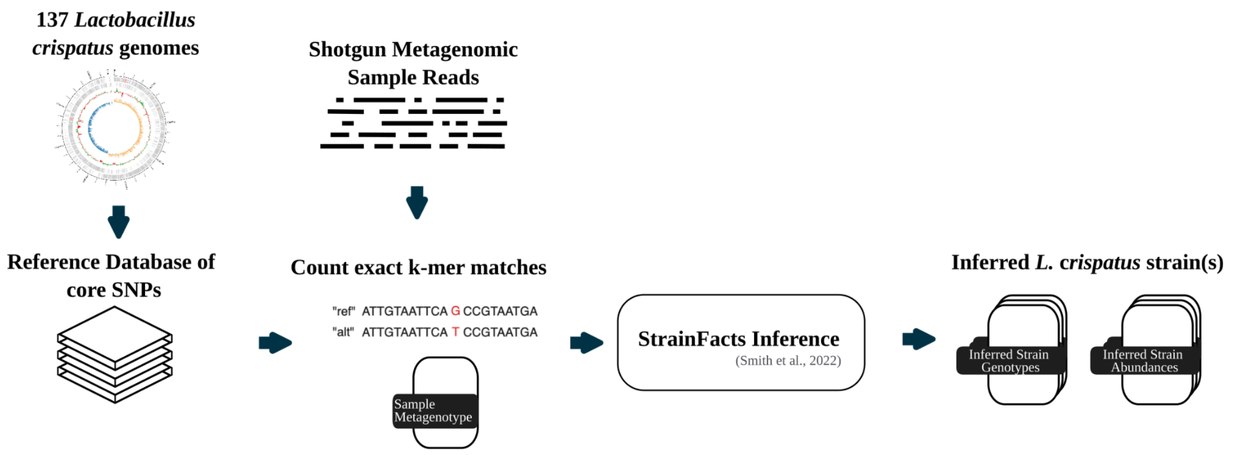

bioRxiv preprint doi: https://doi.org/10.1101/2025.08.15.670563; this version posted August 21, 2025. The copyright holder for this preprint (which was not certified by peer review) is the author/funder, who has granted bioRxiv a license to display the preprint in perpetuity. It is made available under aCC-BY 4.0 International license.

Figure S2. Sequencing depths are associated with metagenotype error

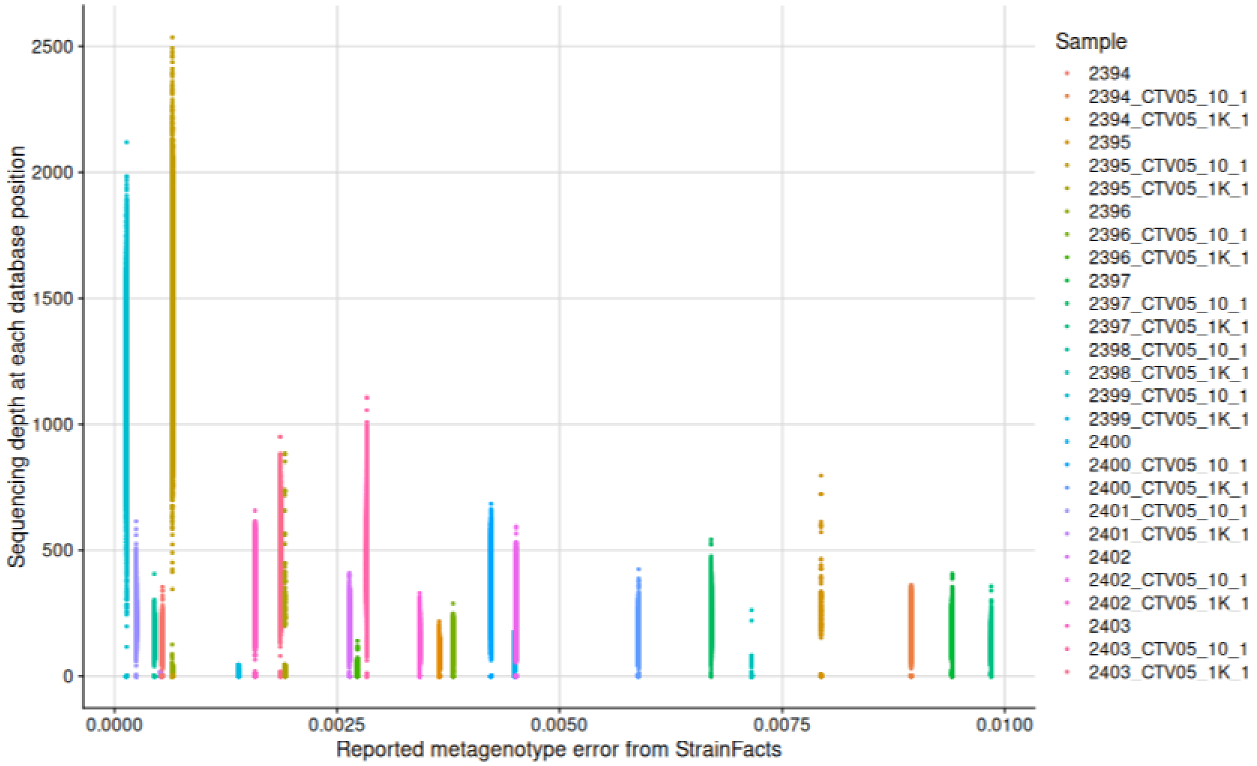

Sequencing depths for each SNV site from a sample with the reported metagenotype errors from StrainFacts. Colors indicate different samples and spike-in concentrations.

bioRxiv preprint doi: https://doi.org/10.1101/2025.08.15.670563; this version posted August 21, 2025. The copyright holder for this preprint (which was not certified by peer review) is the author/funder, who has granted bioRxiv a license to display the preprint in perpetuity. It is made available under aCC-BY 4.0 International license.

Table S1 comparison of StrainFacts with other strain inference methods

Tool	Reference Database	Depth required	Tracks strains across samples	Multiple strains per sample	Strain Composition	Strain Definition
**StrainFacts(1)**	Genome database	Low	Yes	Yes	Relative abundance of strains	Combination of bi-allelic SNVs (de novo)
**inStrain(2)**	Single genome	Low	Yes	No (“Strain cloud” plus allele frequencies)	Allele frequencies (indirect quantification)	popANI-based
**StrainPhlAn(3)**	Species-specific marker genes (MetaPhlAn)	Low	Yes	No (single consensus strain)	Presence/absence (dominant strain)	Reference-based
**StrainGE(4)**	Genome database (e.g., RefSeq)	Low	?	Yes	Presence/absence (with abundance via coverage)	Nearest reference

bioRxiv preprint doi: https://doi.org/10.1101/2025.08.15.670563; this version posted August 21, 2025. The copyright holder for this preprint (which was not certified by peer review) is the author/funder, who has granted bioRxiv a license to display the preprint in perpetuity. It is made available under aCC-BY 4.0 International license.

**Strain Finder(5)**	Single genome	High	Yes	Yes	Relative abundance of strains	de novo
**PStrain(6)**	Species-specific marker genes	High	No	Yes	Relative abundance of strains	Nearest reference

bioRxiv preprint doi: https://doi.org/10.1101/2025.08.15.670563; this version posted August 21, 2025. The copyright holder for this preprint (which was not certified by peer review) is the author/funder, who has granted bioRxiv a license to display the preprint in perpetuity. It is made available under aCC-BY 4.0 International license.

Features of available strain tracking methods. (1) Smith BJ, Li X, Shi ZJ, Abate A, Pollard KS. Scalable microbial strain inference in metagenomic data using StrainFacts. Front Bioinform. 2022;2:867386. FrontiersPubMed (2) Olm MR, Crits-Christoph A, Bouma-Gregson K, Firek BA, Morowitz MJ, Banfield JF. inStrain profiles population microdiversity from metagenomic data and sensitively detects shared microbial strains. Nat Biotechnol. 2021;39(6):727-736. NaturePubMed (3) Truong DT, Tett A, Pasolli E, Huttenhower C, Segata N. Microbial strain-level population structure and genetic diversity from metagenomes. Genome Res. 2017;27(4):626-638. Cold Spring Harbor LaboratoryPubMed Central(4)van Dijk LR, Walker BJ, Straub TJ, Worby CJ, Grote A, Schreiber HL, et al. StrainGE: a toolkit to track and characterize low-abundance strains in complex microbial communities. Genome Biol. 2022;23(1):74. Genome BiologyPubMed (5)Smillie CS, Sauk J, Gevers D, Friedman J, Sung J, Youngster I, et al. Strain tracking reveals the determinants of bacterial engraftment in the human gut following fecal microbiota transplantation. Cell Host Microbe. 2018;23(2):229-240.e5. Principles of microbiota engraftment | Nature Reviews Microbiology (6) Wang S, Jiang Y, Li S. PStrain: an iterative microbial strains profiling algorithm for shotgun metagenomic sequencing data. Bioinformatics. 2020;36(22-23):5499-5506. Oxford AcademicNCBI

bioRxiv preprint doi: https://doi.org/10.1101/2025.08.15.670563; this version posted August 21, 2025. The copyright holder for this preprint (which was not certified by peer review) is the author/funder, who has granted bioRxiv a license to display the preprint in perpetuity. It is made available under aCC-BY 4.0 International license.

Table S2. Number of inferred strains of each true strain across simulated cohorts

	**Cohort 1, N = 226**[1]	**Cohort 2, N = 219**[1]	**Cohort 3, N = 223**[1]	**Cohort 4, N = 225**[1]	**Cohort 5, N = 226**[1]	**Cohort 6, N = 229**[1]	**Cohort 7, N = 218**[1]	**Cohort 8, N = 228**[1]	**Cohort 9, N = 227**[1]	**Cohort 10, N = 218**[1]
0	93 (41%)	104 (47%)	100 (45%)	95 (42%)	92 (41%)	101 (44%)	96 (44%)	110 (48%)	108 (48%)	106 (49%)
1	119 (53%)	108 (49%)	115 (52%)	121 (54%)	126 (56%)	120 (52%)	108 (50%)	107 (47%)	112 (49%)	103 (47%)
2	14 (6.2%)	7 (3.2%)	8 (3.6%)	9 (4.0%)	8 (3.5%)	8 (3.5%)	14 (6.4%)	11 (4.8%)	7 (3.1%)	9 (4.1%)

[1] n (%)

Number (percent) of inferred strains that genotypically match each true strain with Jaccard similarity >0.9 for each of the 10 simulated cohorts (number of strains analyzed per cohort is indicated in the header of each column). Analysis is restricted to true strains with ≥10% abundance in at least one sample.

bioRxiv preprint doi: https://doi.org/10.1101/2025.08.15.670563; this version posted August 21, 2025. The copyright holder for this preprint (which was not certified by peer review) is the author/funder, who has granted bioRxiv a license to display the preprint in perpetuity. It is made available under aCC-BY 4.0 International license.

Table S3. Percentage of well-matched inferred strains that are near identical

Simulated Cohort	% of true strains with well-matching pairs of inferred that are near identical (Jaccard Similarity > 0.99)
1	71.43%
2	92.86%
3	81.25%
4	88.89%
5	75.00%
6	81.25%
7	67.86%
8	77.27%
9	78.57%
10	88.89%

Percentage of true strains with two well-matching inferred strains (>0.9 Jaccard similarities of each inferred strain to the true strain) in which the pair of inferred strains were nearly identical to each other (>0.99 Jaccard similarity between the inferred strains).
